# Walking the line: Does crossing a high stakes exam threshold matter for labour market outcomes?

**DOI:** 10.23889/ijpds.v8i2.2187

**Published:** 2023-09-14

**Authors:** Oliver Anderson

**Affiliations:** 1 Department for Education, London, United Kingdom

## Objectives

This paper offers new insight into the link between success in high stakes exams and subsequent education and labour market outcomes. It is the first study to look holistically at the impact of crossing an important high stakes threshold on both academic and vocational education choices and ultimately labour market outcomes.

## Method

It does so by comparing those either side of a formerly important threshold in the English education system at the end of compulsory schooling (achieving five general certificate of secondary education A* to C passes) which was commonly regarded as the minimum benchmark for continuing into post-compulsory education.

## Results

I find that crossing this threshold led to an 6.3-6.7 percentage point increase in the proportion of men and women (respectively) going on to take academic qualifications, with little change in the proportion taking vocational qualifications, leading to a net increase in those staying on after compulsory schooling. Women's daily earnings in 2017-18 (11-13 years after leaving compulsory schooling) were 3.1 percentage points higher for those just crossing the threshold, but men's early labour market outcomes were unchanged.

## Conclusion

The results for men can be explained by low returns to academic qualifications for marginal learners. The findings for women do not disappear after accounting for subsequent education choices, suggesting that crossing the threshold may play a signalling role for employers as well as education institutions.

